# Supplementary material for: Parasitoid biology preserved in mineralized fossils
Source: Nat Commun. 2018 Aug 28;9:3325. doi: 10.1038/s41467-018-05654-y (PMC6113268; doi:10.1038/s41467-018-05654-y)
Supplement: Supplementary file 1 — Supplementary Information [file 41467_2018_5654_MOESM1_ESM.pdf]

# Supplementary Information

# Parasitoid biology preserved in mineralized fossils

van de Kamp *et al.*

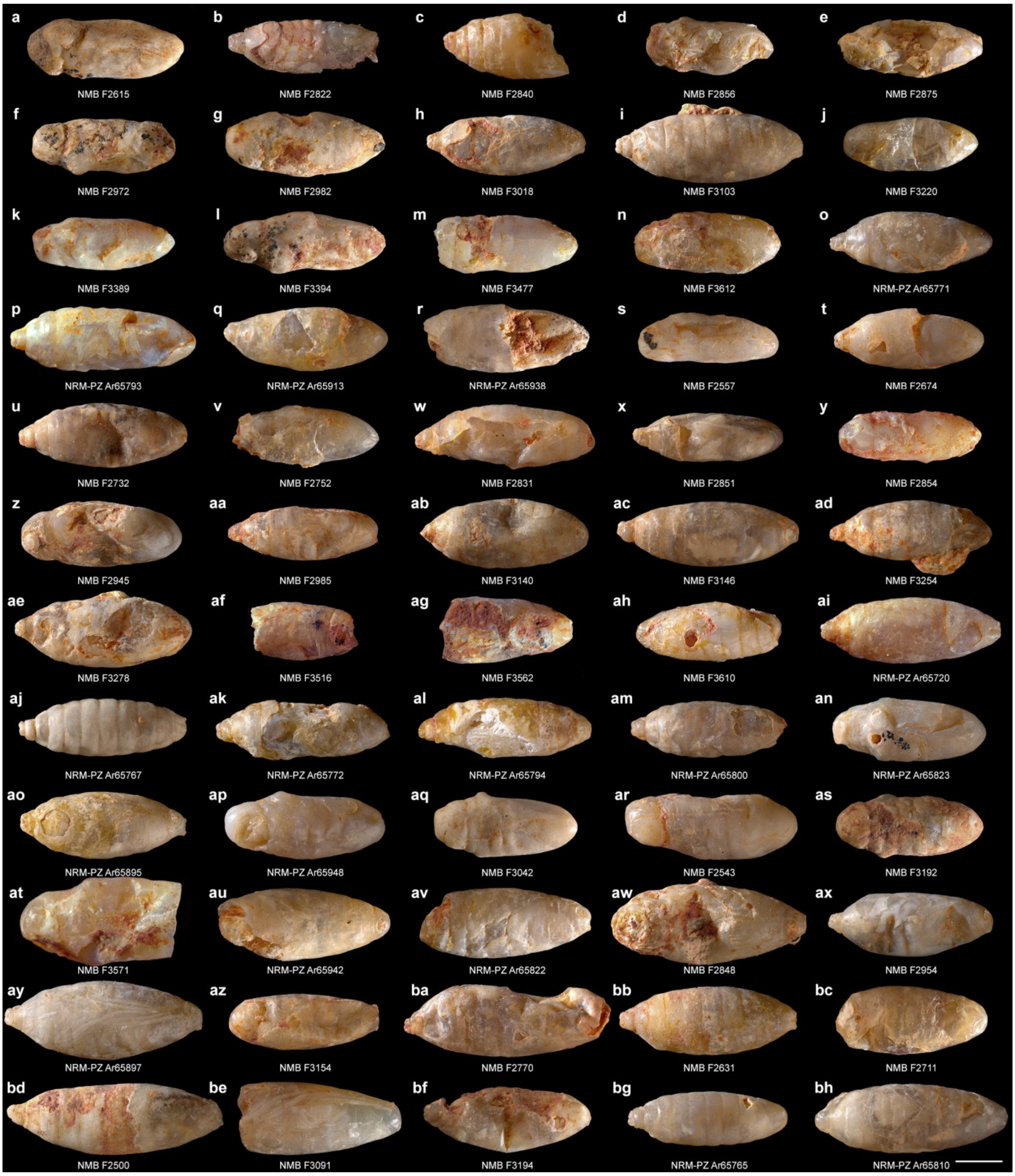


**Supplementary Figure 1 | Photographs of the fossils, in which the following parasitation events or host remains were identified: a-r:** *Xenomorphia resurrecta* females; **s-ap:** *X. resurrecta* males; **aq:** *X. handschini* female; **ar-au:** *X. handschini* males; **av:** *X. handschini* pupa of unknown sex; **aw-ay:** *Coptera anka* females; **az:** *C. anka* male; **ba:** *Palaeortona quercyensis* female; **bb:** unidentified putative second instar larva; **bc:** last-instar larval mandibles left-behind by an emerged parasitoid; **bd-bh:** preserved parts of the host flies. Scale bar: 1 mm.

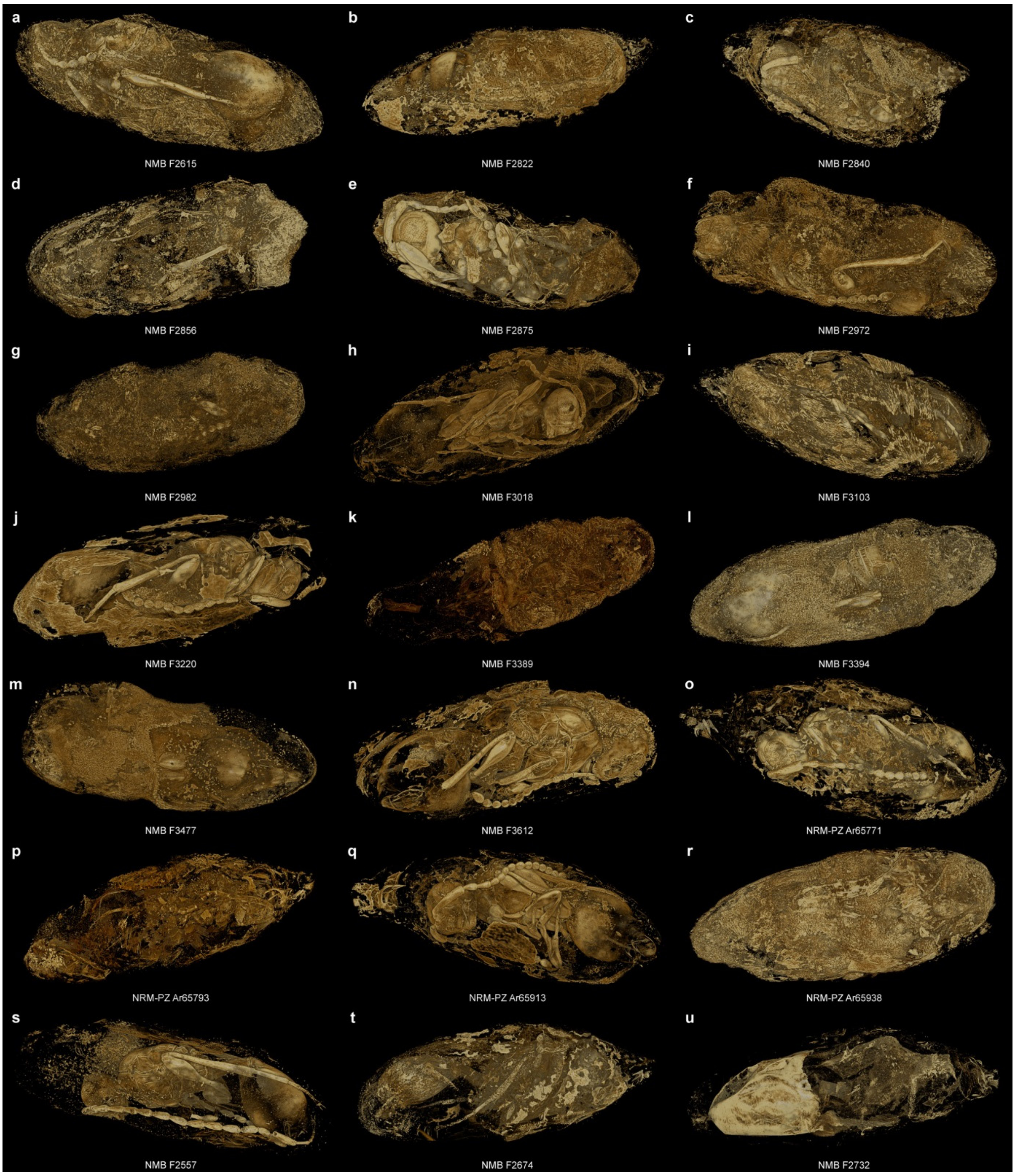


**Supplementary Figure 2 | Volume renderings of the inside of *Eophora* pupae, in which parasitation events were identified (perspective view). a-r:** *Xenomorphia resurrecta* females; **s-u:** *X. resurrecta* males. See Supplementary Fig. 1 for external shape of the puparia and scale bar.

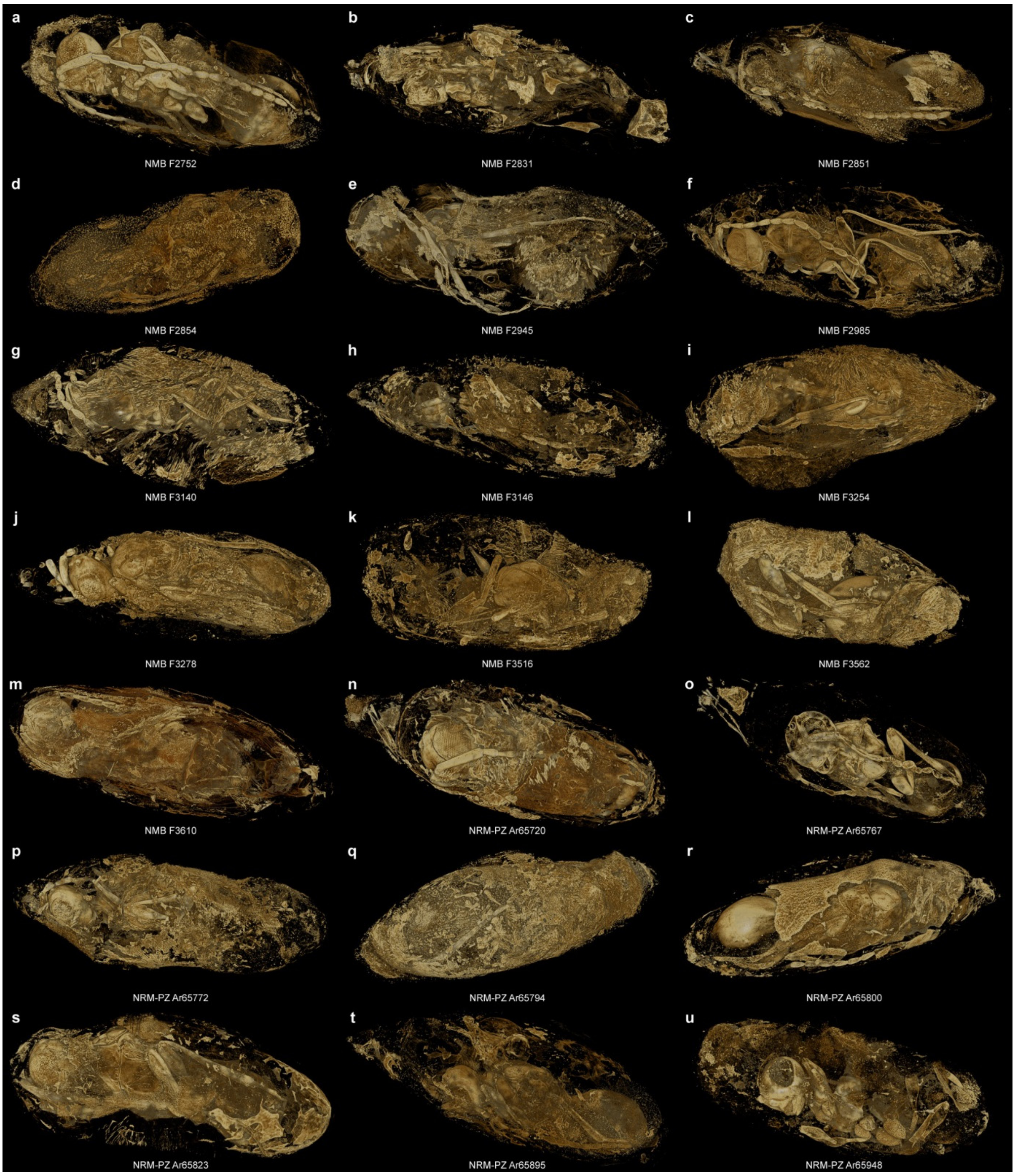


**Supplementary Figure 3 | Volume renderings of the inside of *Eophora* pupae, in which parasitation events were identified (*Xenomorphia resurrecta* males; perspective view).** See Supplementary Fig. 1 for external shape of the puparia and scale bar.

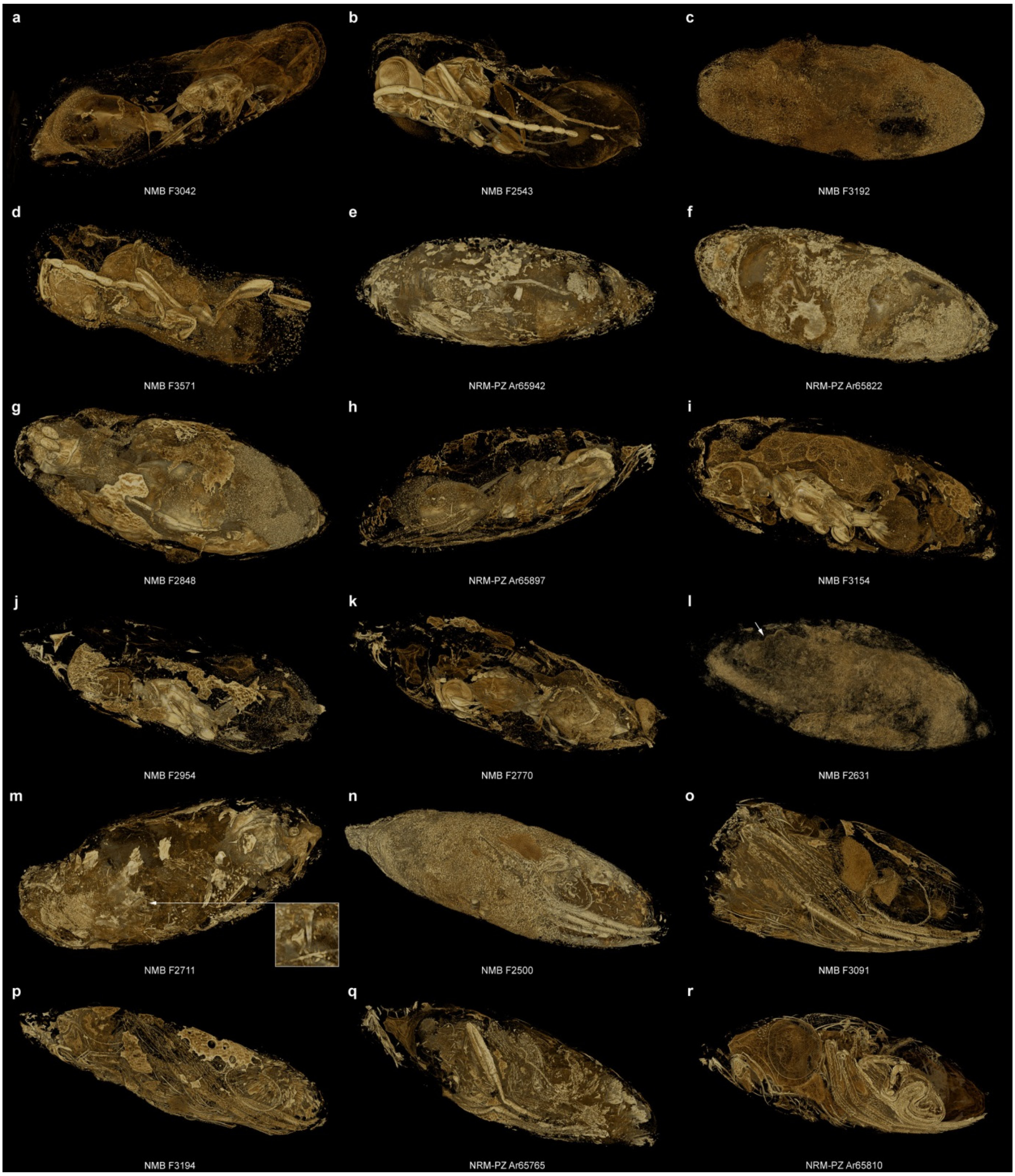


**Supplementary Figure 4 | Volume renderings (perspective view) of the inside of *Eophora* pupae, in which parasitation events or host remains were identified (perspective view). a:** *Xenomorphia handschini* female; **b-e:** *X. handschini* males; **f:** *X. handschini* pupa of unknown sex; **g-i:** *C. anka* females; note the preserved fly host legs in (h); **j:** *C. anka* male; **k:** *Palaeortona quercyensis* female; **l:** unidentified putative second instar larva (arrow); **m:** last-instar larval mandibles left-behind by an emerged parasitoid (see inset); **n-r:** preserved parts of the host flies. See Supplementary Fig. 1 for external shape of the puparia and scale bar.

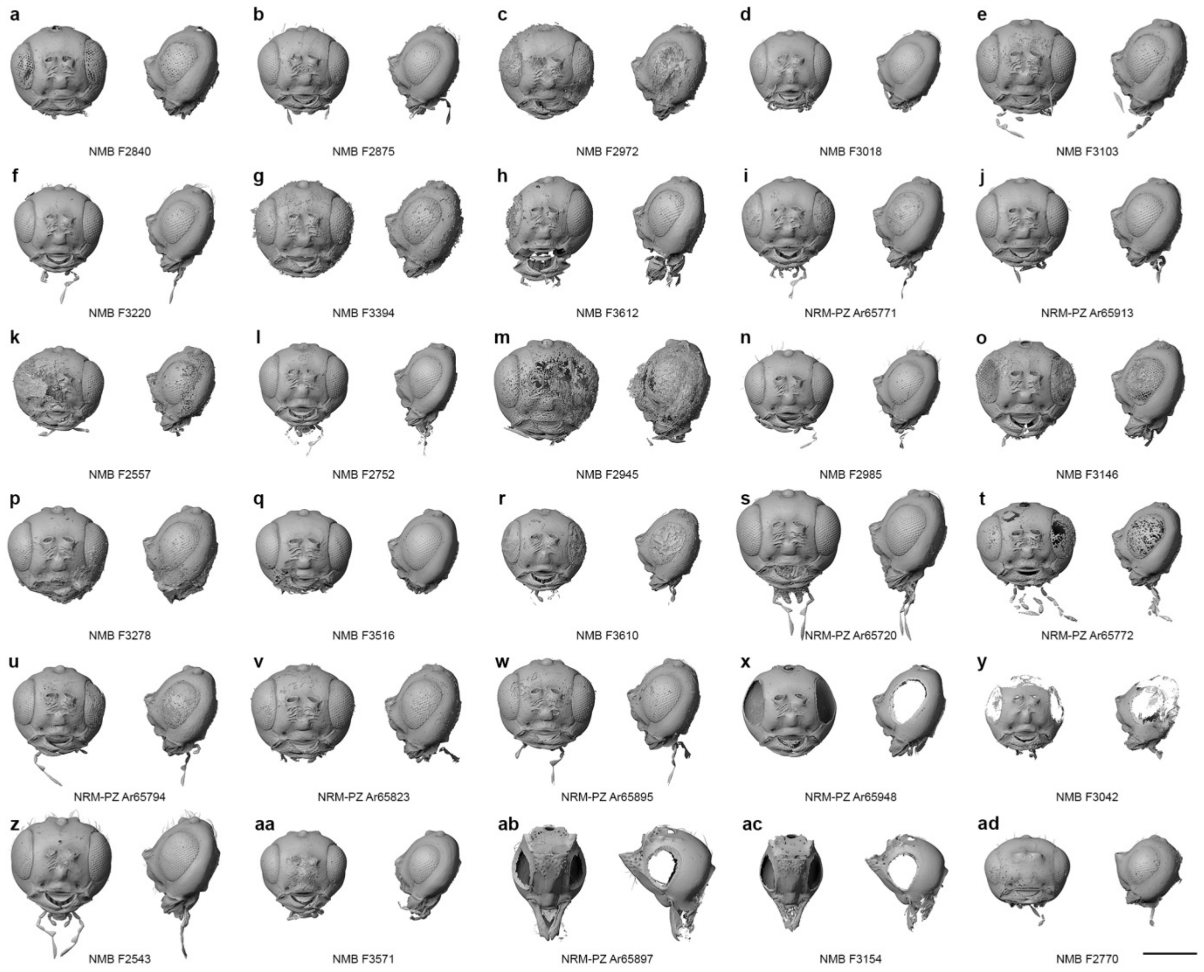


**Supplementary Figure 5 | Surface renderings of 30 parasitoid heads. a-j:** *Xenomorphia resurrecta* females; **k-x:** *X. resurrecta* males; **y:** *X. handschini* female; **z,aa:** *X. handschini* males; **ab:** *Coptera anka* female; **ac:** *C. anka* male; **ad:** *Palaeortona quercyensis* female. Scale bar: 250 µm.

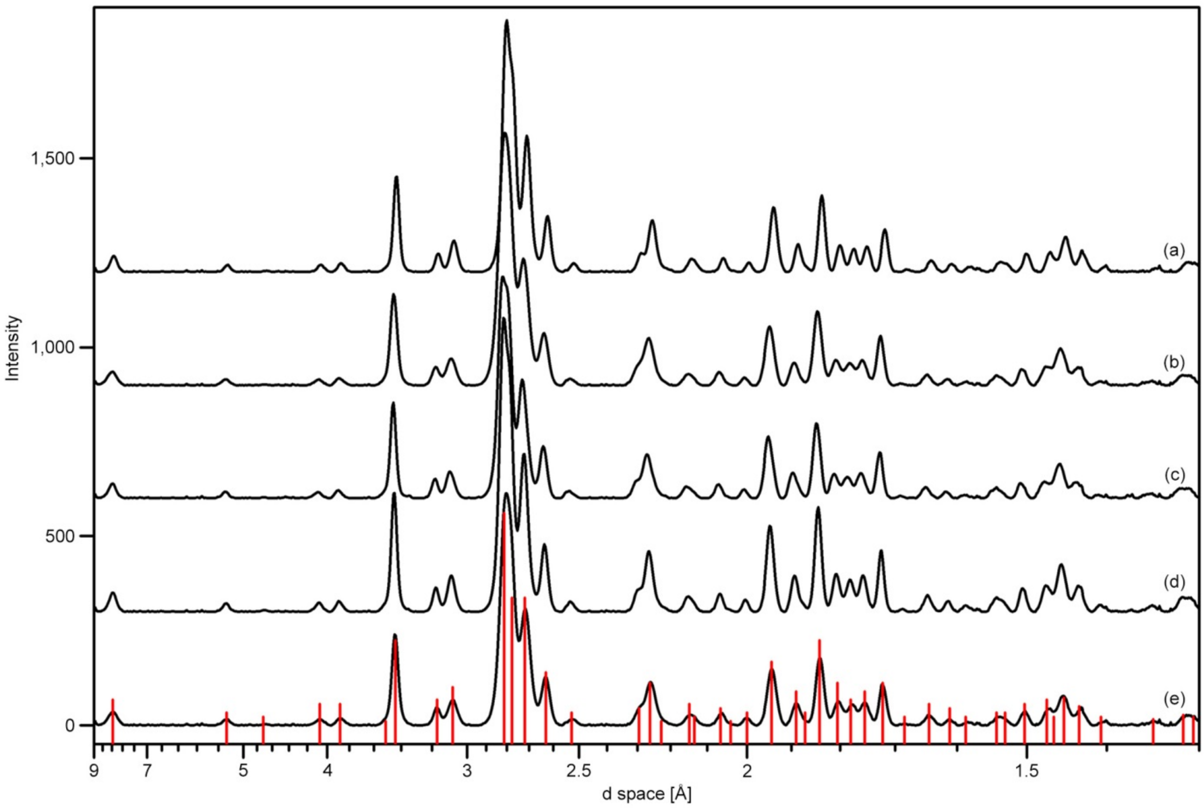


**Supplementary Figure 6 | Synchrotron X-ray diffraction pattern of five pupae**. NMB F2459 (a), NMB F2460 (b), NMB F2531 (c), NMB F2557 (d) & NMB F2915 (e). All samples consist of the mineral apatite (general formula $Ca_5(PO_4)_3(OH,F,Cl)$, indicated by a line pattern from the ICDD database (entry PDF 9-432).

## Supplementary Table 1 | List of identified parasitation events

| collection no. | species | sex | stage | posture | wings | reference length (µm) | external preservation of the host | preservation of the parasitoid |
|---|---|---|---|---|---|---|---|---|
| NMB F2615 | *X. resurrecta* | female | adult | asymmetric | unfolded | 911 | pupal skin only | generally well-preserved; mesosoma damaged dorsally |
| NMB F2822 | *X. resurrecta* | female | adult | symmetric | folded | 785 | puparium largely detached from pupal skin | generally well-preserved; head hollow |
| NMB F2840 | *X. resurrecta* | female | adult | symmetric | folded | 826 | posterior half of puparium missing | generally well-preserved and hollow; posterior part of gaster missing |
| NMB F2856 | *X. resurrecta* | female | adult | asymmetric | unfolded | 790 | pupal skin only; antenna shines through | generally poorly preserved; head damaged |
| NMB F2875 | *X. resurrecta* | female | adult | asymmetric | unfolded | 824 | pupal skin only | well-preserved; remnants of flight muscles visible |
| NMB F2972 | *X. resurrecta* | female | adult | symmetric | unfolded | 890 | pupal skin only | generally well-preserved; head and legs partly damaged |
| NMB F2982 | *X. resurrecta* | female | adult | asymmetric | unfolded | 851 | puparium largely detached from pupal skin | generally poorly preserved and partly connected to adjacent voids; head capsule damaged |
| NMB F3018 | *X. resurrecta* | female | adult | asymmetric | unfolded | 765 | complete puparium; slightly damaged | well-preserved; antennae and legs largely hollow |
| NMB F3103 | *X. resurrecta* | female | adult | asymmetric | unfolded | 941 | complete puparium; small piece of stony matrix attached | generally well-preserved; head capsule hollow |
| NMB F3220 | *X. resurrecta* | female | adult | symmetric | folded | 772 | pupal skin only | well-preserved; head capsule largely hollow |
| NMB F3389 | *X. resurrecta* | female | adult | symmetric | not preserved | 779 | pupal skin only; damaged | generally poorly preserved; head and gaster deformed |
| NMB F3394 | *X. resurrecta* | female | adult | asymmetric | not preserved | 934 | pupal skin only | generally well-preserved but partly connected to adjacent voids |
| NMB F3477 | *X. resurrecta* | female | adult | ? | not preserved | 817 | puparium largely detached from pupal skin; anterior part missing | poorly preserved and partly connected to adjacent voids; head missing; gaster deformed |
| NMB F3612 | *X. resurrecta* | female | adult | asymmetric | unfolded | 847 | pupal skin only; slightly damaged | generally well-preserved; head, mesopectus, gaster and legs slightly damaged |
| NRM-PZ Ar65771 | *X. resurrecta* | female | adult | symmetric | folded | 813 | complete puparium | well-preserved; head capsule largely hollow |
| NRM-PZ Ar65793 | *X. resurrecta* | female | adult | ? | not preserved | 895 | puparium partly detached from pupal skin | generally poorly preserved; petiole barely recognizable; gaster deformed |
| NRM-PZ Ar65913 | *X. resurrecta* | female | adult | asymmetric | unfolded | 824 | puparium slightly damaged and posteriorly detatched from pupal skin | well-preserved; petiole hollow |
| NRM-PZ Ar65938 | *X. resurrecta* | female | adult | asymmetric | not preserved | 938 | puparium posteriorly detached from pupal skin; pieces of stony matrix attached | poorly preserved and partly connected to adjacent voids |
| NMB F2557 | *X. resurrecta* | male | adult | symmetric | folded | 836 | pupal skin only | generally well-preserved; head hollow and damaged anteriorly |
| NMB F2674 | *X. resurrecta* | male | adult | asymmetric | unfolded | 857 | puparium posteriorly detached from pupal skin | generally poorly preserved; legs of host fly partly visible |
| NMB F2732 | *X. resurrecta* | male | adult | ? | not preserved | 835 | complete puparium | generally poorly preserved and heavily deformed; petiole distinct |
| NMB F2752 | *X. resurrecta* | male | adult | asymmetric | folded | 765 | puparium largely detached from pupal skin | generally well-preserved; left profemur deformed; left antenna broken; remnants of flight muscles visible |
| NMB F2831 | *X. resurrecta* | male | adult | symmetric | folded | 811 | puparium partly detached from pupal skin | poorly preserved; head and gaster deformed |
| NMB F2851 | *X. resurrecta* | male | adult | symmetric | folded | 763 | puparium anteriorly detached; antenna of parasitoid visible through pupal skin | head barely recognizable; mesonotum well-preserved; mesopectus barely recognizable; petiole well-preserved; gaster deformed |
| NMB F2854 | *X. resurrecta* | male | adult | ? | not preserved | 901 | pupal skin only | poorly preserved and hardly traceable |
| NMB F2945 | *X. resurrecta* | male | adult | asymmetric | unfolded | 943 | pupal skin only; anteriorly damaged | generally well-preserved; head detached; mesosoma and petiole well-preserved; gaster barely recognizable |
| NMB F2985 | *X. resurrecta* | male | adult | asymmetric | unfolded | 774 | pupal skin only | generally well-preserved; left antenna broken; left metatibia detached |
| NMB F3140 | *X. resurrecta* | male | adult | asymmetric | unfolded | 816 | complete puparium | generally well-preserved; head capsule barely recognizable; several antennal segments and left mesofemur detached |
| NMB F3146 | *X. resurrecta* | male | adult | symmetric | folded | 871 | complete puparium | generally poorly preserved and partly connected to adjacent voids; head detached; compound eyes barely recognizable; posterior mesosoma poorly preserved |
| NMB F3254 | *X. resurrecta* | male | adult | symmetric | folded | 804 | complete puparium; small piece of stony matrix attached | generally well-preserved but partly connected to adjacent voids; compound eyes barely |

| | | | | | | | | |
|---|---|---|---|---|---|---|---|---|
| | | | | | | | | recognizable |
| NMB F3278 | *X. resurrecta* | male | adult | asymmetric | unfolded | 894 | puparium partly damaged; small piece of stony matrix attached | generally well-preserved; several antennal segments detached |
| NMB F3516 | *X. resurrecta* | male | adult | ? | not preserved | n/a | puparium damaged anteriorly and posteriorly; endocast of parasitoid's gaster visible | generally well-preserved but collapsed into pieces |
| NMB F3562 | *X. resurrecta* | male | adult | symmetric | folded | n/a | puparium heavily damaged; anterior part missing | generally poorly preserved; head and anterior mesosoma missing; ventrally damaged |
| NMB F3610 | *X. resurrecta* | male | adult | symmetric | folded | 761 | puparium partly detached from pupal skin | generally well-preserved; gaster damaged dorsally |
| NRM-PZ Ar65720 | *X. resurrecta* | male | adult | symmetric | folded | 911 | complete puparium; posterior top slightly damaged | well-preserved; head hollow |
| NRM-PZ Ar65767 | *X. resurrecta* | male | adult | symmetric | folded | 832 | complete puparium | generally poorly preserved; head deformed; compound eyes missing; legs and metapectal-propodeal complex hardly recognizable |
| NRM-PZ Ar65772 | *X. resurrecta* | male | adult | asymmetric | unfolded | 777 | puparium partly detached from pupal skin; endocast of the parasitoid's gaster visible | generally well-preserved but partly connected to adjacent voids; antennae and legs partly collapsed |
| NRM-PZ Ar65794 | *X. resurrecta* | male | adult | asymmetric | unfolded | 871 | puparium damaged | poorly preserved and partly connected to adjacent voids; head hollow |
| NRM-PZ Ar65800 | *X. resurrecta* | male | adult | symmetric | folded | 786 | complete puparium with posteriorly damaged surface | generally well-preserved; head barely recognizable |
| NRM-PZ Ar65823 | *X. resurrecta* | male | adult | asymmetric | unfolded | 896 | pupal skin only | well-preserved; head, mesosoma and petiole hollow |
| NRM-PZ Ar65895 | *X. resurrecta* | male | adult | symmetric | folded | 782 | complete puparium; circular anteroventral damage | generally well-preserved but partly connected to adjacent voids |
| NRM-PZ Ar65948 | *X. resurrecta* | male | adult | asymmetric | not preserved | 856 | pupal skin only | generally well-preserved; compound eyes missing; parts of mesosoma barely recognizable; gaster damaged |
| NMB F3042 | *X. handschini* | female | adult | symmetric | folded | 734 | pupal skin only; right antenna and procoxae visible | mandibles, dorsal mesosoma and petiole well-preserved; ventral mesosoma barely recognizable; gaster poorly preserved |
| NMB F2543 | *X. handschini* | male | adult | symmetric | folded | 911 | pupal skin only; left antenna visible | head and anterior mesosoma well-preserved; posterior mesosoma barely recognizable; petiole and gaster poorly preserved |
| NMB F3192 | *X. handschini* | male | adult | ? | not preserved | n/a | pupal skin only | generally poorly preserved and largely connected to adjacent voids; petiole and parts of mesosoma well recognizable; remnants of flight muscles visible |
| NMB F3571 | *X. handschini* | male | adult | asymmetric | unfolded | 817 | pupal skin only; posterior third missing | generally well-preserved; petiole and gaster hollow; antennae and legs partly deformed; posterior part of gaster missing |
| NRM-PZ Ar65942 | *X. handschini* | male | adult | asymmetric | not preserved | n/a | puparium; damaged anteriorly | generally poorly preserved; only some body parts recognizable (e.g. head capsule, petiole, gaster) |
| NRM-PZ Ar65822 | *X. handschini* | unknown | pupa | n/a | n/a | n/a | puparium; damaged anteriorly | poorly preserved and deformed; largely connected to adjacent voids; species identification based on broad petiole |
| NMB F2848 | *C. anka* | female | adult | asymmetric | unfolded | 794 | pupal skin only | head and anterior mesosoma apparently cut off; otherwise well-preserved |
| NMB F2954 | *C. anka* | female | adult | symmetric | folded | 703 | complete puparium; slightly dented | head poorly preserved; compound eyes missing; mesosoma and petiole well-preserved, gaster poorly preserved; distal parts of antennae and legs barely recognizable |
| NRM-PZ Ar65897 | *C. anka* | female | adult | symmetric | folded | 717 | complete puparium; dented; fly legs visible | generally well-preserved; compound eyes missing; distal parts of antennae and legs barely recognizable; legs of host fly partly visible |
| NMB F3154 | *C. anka* | male | adult | symmetric | not preserved | 652 | pupal skin only | generally well-preserved; compound eyes missing; distal parts of antennae and legs barely recognizable |
| NMB F2770 | *P. quercyensis* | female | adult | asymmetric | unfolded | 810 | complete puparium; dented and slightly damaged | generally well-preserved; only some antennal segments missing |
| NMB F2631 | unknown | unknown | putative 2nd instar larva | n/a | n/a | n/a | complete puparium | preserved as void; fine details like mandibles and hairs missing |
| NMB F2711 | unknown | unknown | larval mandibles | n/a | n/a | n/a | pupal skin and minor parts of puparium | only mandibles of last larval instar |
