# Supplementary material for: Parasitoid biology preserved in mineralized fossils
Source: Nat Commun. 2018 Aug 28;9:3325. doi: 10.1038/s41467-018-05654-y (PMC6113268; doi:10.1038/s41467-018-05654-y)
Supplement: Supplementary file 3 — Description of Additional Supplementary Files [file 41467_2018_5654_MOESM3_ESM.pdf]

Description of Additional Supplementary Files

File Name: Supplementary Movie 1
Description: Isolating a parasitoid from a fossilized puparium. Virtual cut of a mineralized fly puparium (NRM-PZ Ar65720) and digital isolation of the concealed parasitoid wasp (volume rendering).

File Name: Supplementary Movie 2
Description: From tomography data to illustration. The surface reconstruction of a female *Xenomorphia resurrecta* (NMB F2875) is rearranged and artificially colored in order a to provide a true-to-life impression of the specimen, which is shown ovipositing into a puparium (derived from NRM-PZ Ar65767).

File Name: Supplementary Data 1
Description: 3D PDF of female *Xenomorphia resurrecta*. Interactive 3D surface model of a *X. resurrecta* female (NMB F2875). Click on the figure to start interactive 3D view; switch between views by using the menu (Adobe Reader required).

File Name: Supplementary Data 2
Description: 3D PDF of male *Xenomorphia resurrecta*. Interactive 3D surface model of a *X. resurrecta* male (NRM-PZ Ar65720) including host parts. Click on the figure to start interactive 3D view; switch between views by using the menu (Adobe Reader required).

File Name: Supplementary Data 3
Description: 3D PDF of female *Xenomorphia handschini*. Interactive 3D surface model of a *X. handschini* female (NMB F3042). Click on the figure to start interactive 3D view; switch between views by using the menu (Adobe Reader required).

File Name: Supplementary Data 4
Description: 3D PDF of male *Xenomorphia handschini*. Interactive 3D surface model of a *X. handschini* male (NMB F2543). Click on the figure to start interactive 3D view; switch between views by using the menu (Adobe Reader required).

File Name: Supplementary Data 5
Description: 3D PDF of female *Coptera anka*. Interactive 3D surface model of a *C. anka* female (NRM-PZ Ar65897). Click on the figure to start interactive 3D view; switch between views by using the menu (Adobe Reader required).

File Name: Supplementary Data 6
Description: 3D PDF of male *Coptera anka*. Interactive 3D surface model of a *C. anka* male (NMB F3154). Click on the figure to start interactive 3D view; switch between views by using the menu (Adobe Reader required).

File Name: Supplementary Data 7
Description: 3D PDF of female *Palaeortona quercyensis*. Interactive 3D surface model of a *P. quercyensis* female (NMB F2770). Click on the figure to start interactive 3D view; switch between views by using the menu (Adobe Reader required).
