# Supplementary figures and images for: Parasitoid biology preserved in mineralized fossils
Source: Nat Commun. 2018 Aug 28;9:3325. doi: 10.1038/s41467-018-05654-y (PMC6113268; doi:10.1038/s41467-018-05654-y)

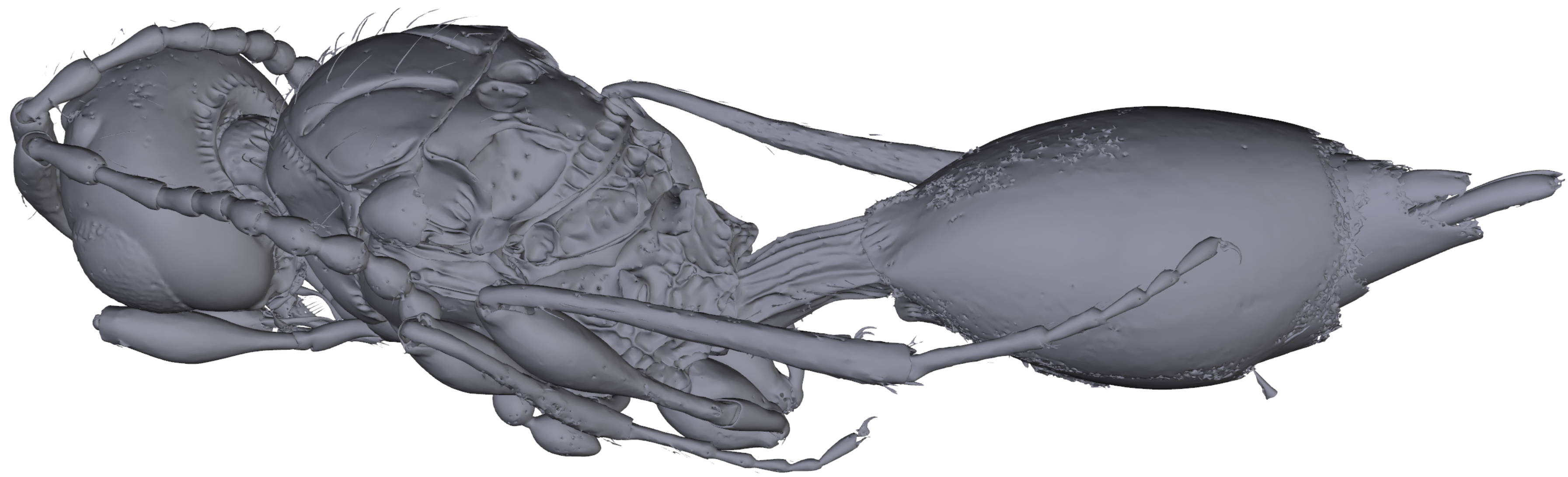

1 mm

Supplement: Supplementary file 6 — Supplementary Data 1 [file 41467_2018_5654_MOESM6_ESM.pdf]

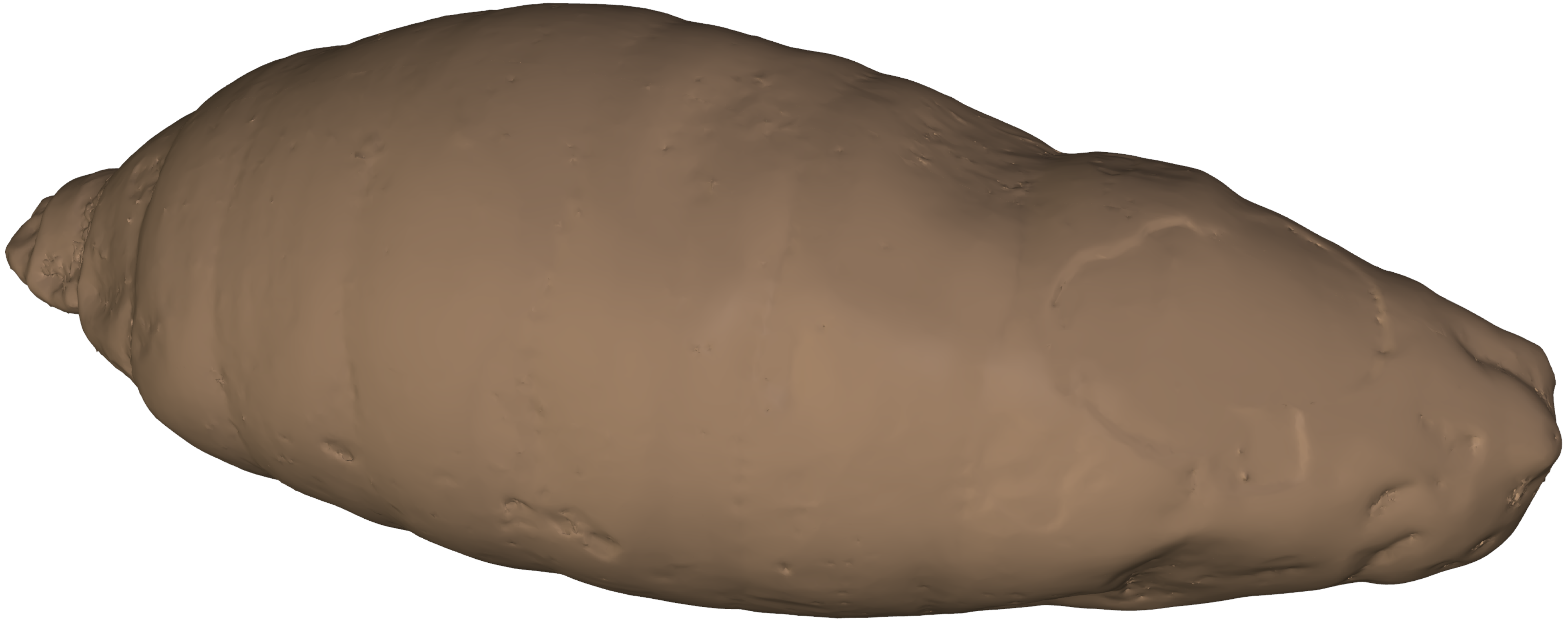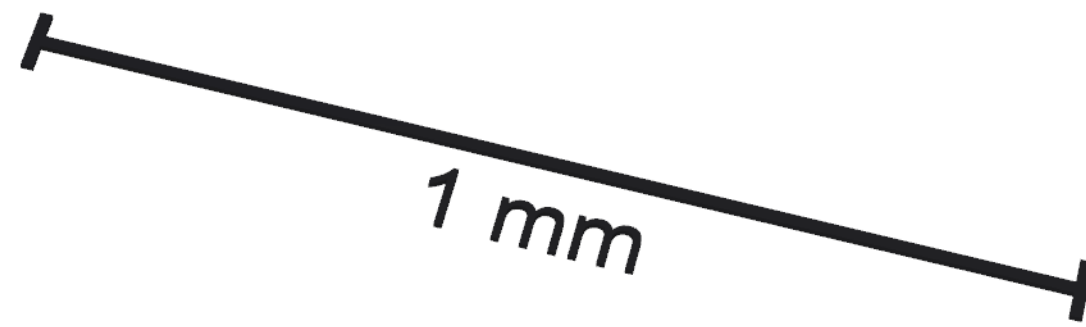

Supplement: Supplementary file 7 — Supplementary Data 2 [file 41467_2018_5654_MOESM7_ESM.pdf]

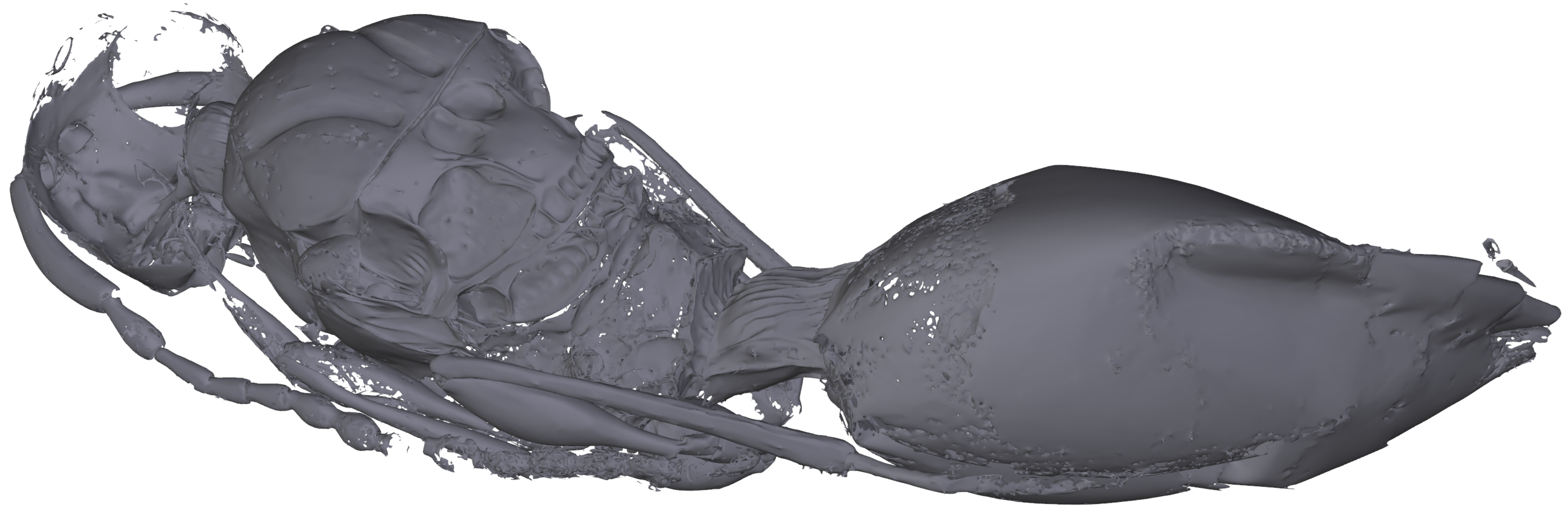

1 mm

Supplement: Supplementary file 8 — Supplementary Data 3 [file 41467_2018_5654_MOESM8_ESM.pdf]

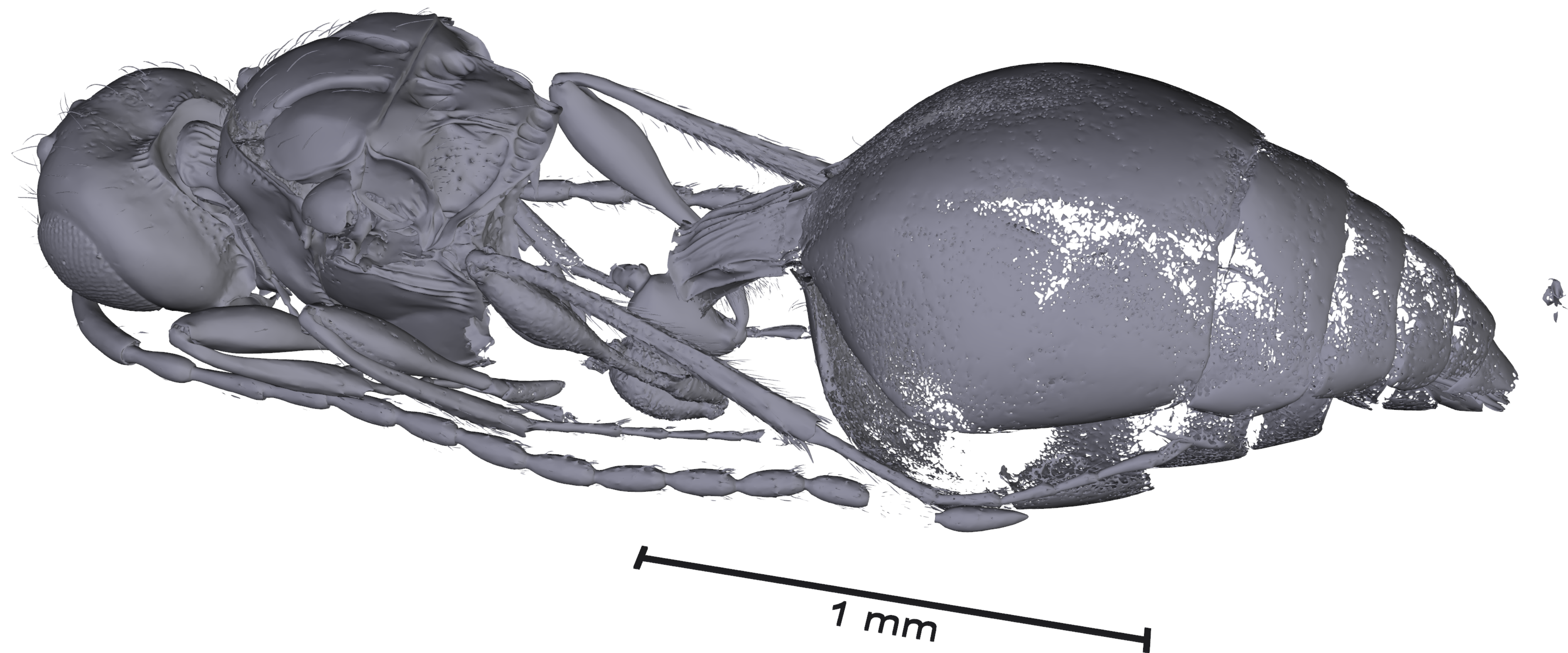

Supplement: Supplementary file 9 — Supplementary Data 4 [file 41467_2018_5654_MOESM9_ESM.pdf]

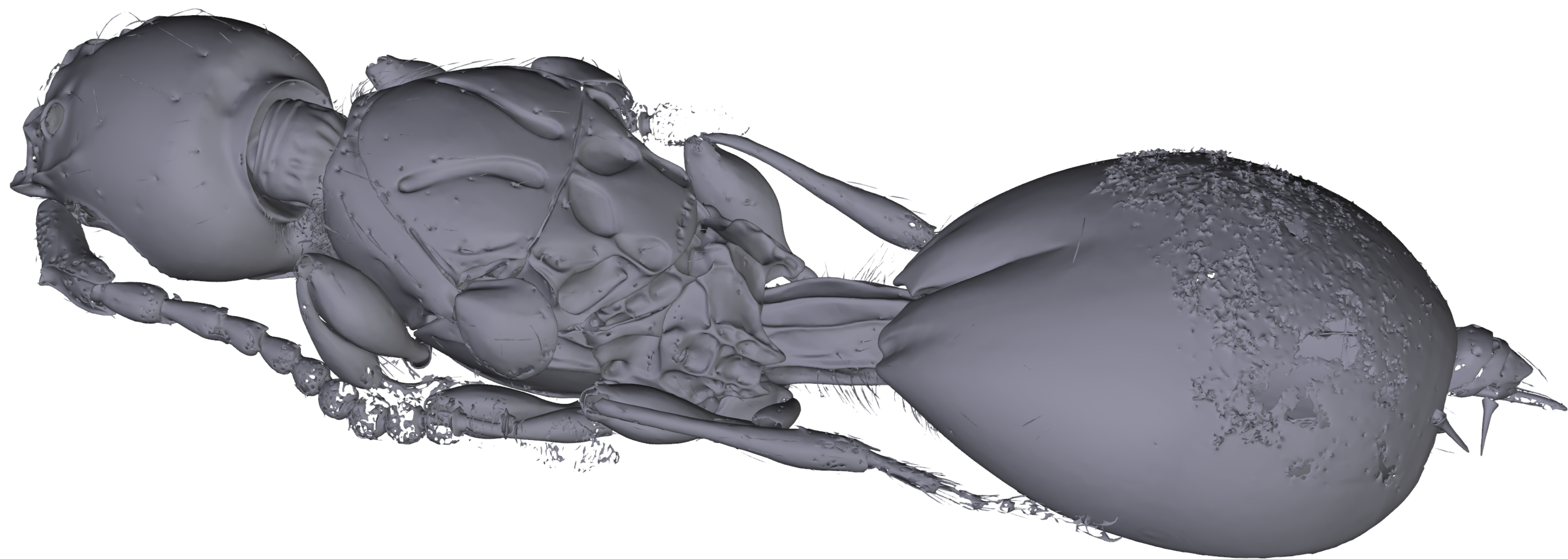

1 mm

Supplement: Supplementary file 10 — Supplementary Data 5 [file 41467_2018_5654_MOESM10_ESM.pdf]

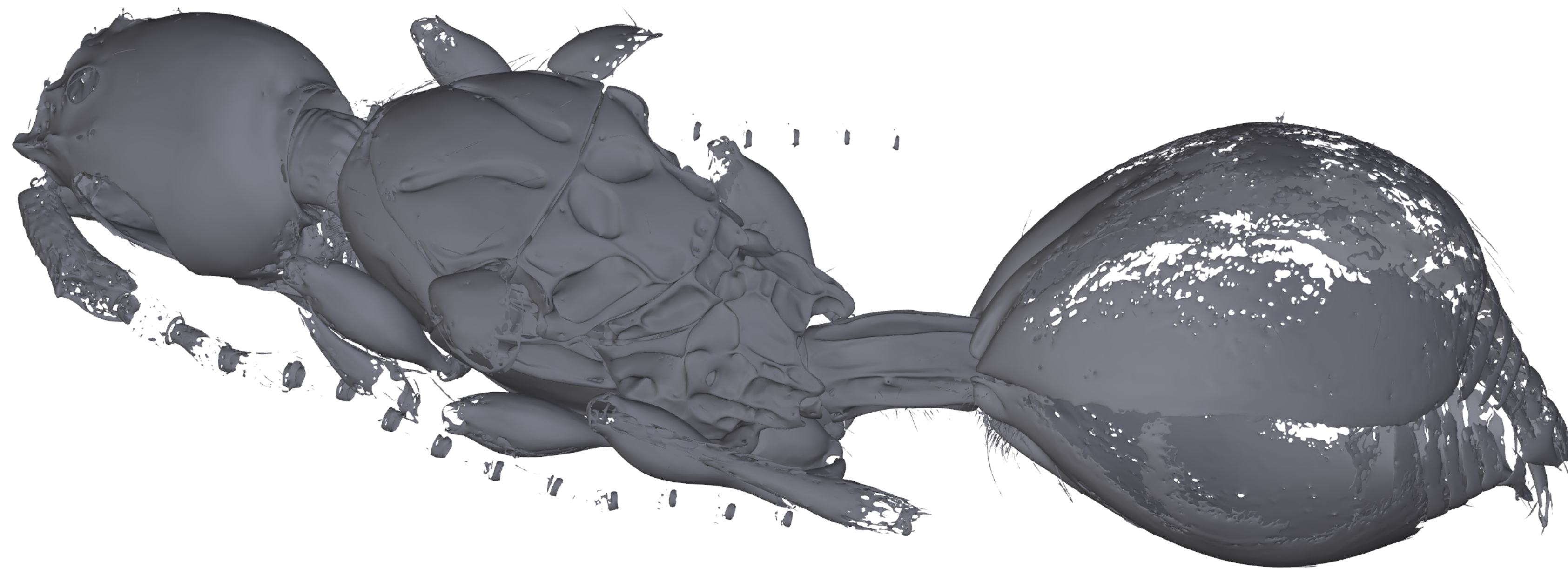

1 mm

Supplement: Supplementary file 11 — Supplementary Data 6 [file 41467_2018_5654_MOESM11_ESM.pdf]

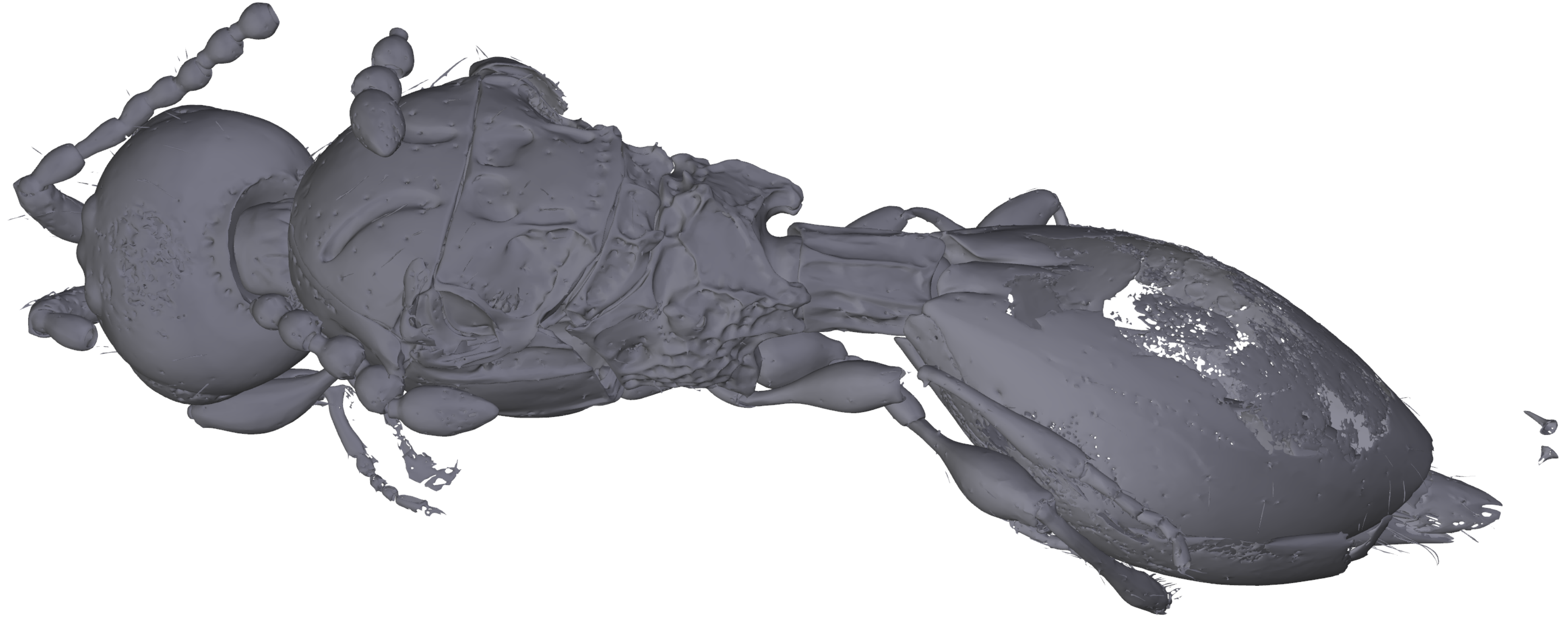

1 mm

Supplement: Supplementary file 12 — Supplementary Data 7 [file 41467_2018_5654_MOESM12_ESM.pdf]
